# Supplementary figures and images for: Construction of a high-density linkage map and detection of sex-specific markers in Penaeus japonicus
Source: PeerJ. 2021 Oct 29;9:e12390. doi: 10.7717/peerj.12390 (PMC8559604; doi:10.7717/peerj.12390)

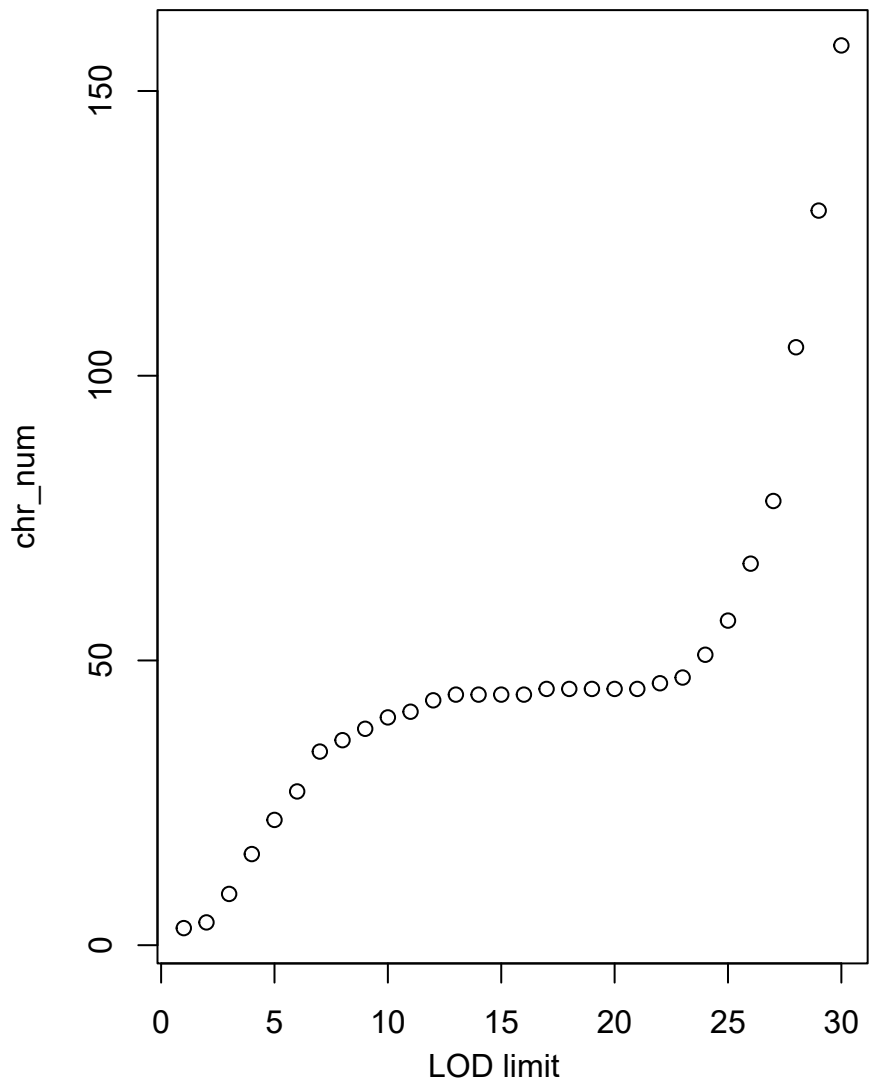

Supplement: Supplemental Information 5 [file peerj-09-12390-s005.pdf]
